# The scale-up finance gap in the EU: Causes, consequences, and policy solutions

**DOI:** 10.1016/j.emj.2022.08.003

**Published:** 2022-10

**Authors:** Anita Quas, Colin Mason, Ramón Compañó, Giuseppina Testa, James P. Gavigan

**Affiliations:** a University of Milan, Italy; b University of Glasgow, United Kingdom; c European Commission, Joint Research Centre, Spain; d University of Foggia, Italy; e European Commission, Joint Research Centre, Belgium

**Keywords:** Scale-up, Scale-up gap, Policy design, Venture capital, European union

## Abstract

This paper assesses the financing gap faced by innovative start-ups in the European Union (EU) when they reach the scaling-up stage of their development. It draws on data collected through a seminar involving 117 experts representing start-ups and scale-ups, financial actors, academia, and EU policymakers and aimed at investigating the causes, consequences, and policy solutions of the scale-up gap in the EU. Besides highlighting supply-side deficiencies, the seminar also emphasised weaknesses in both the demand side of the scale-up gap and the EU ecosystem for high-growth entrepreneurship. The paper offers policy recommendations, highlighting the need for policy solutions involving private actors and specifically targeting scale-ups. The paper also calls for more robust research on measuring the scale-up financing gap and its economic impact to improve the policy response.

## 1 Introduction

Europe underperforms with respect to other major economies, such as the USA, in the creation of scale-ups, i.e. entrepreneurial ventures that are ‘are entering a growth phase where they seek significant market penetration’ (Duruflé et al., 2018, p. 179; Moules, 2021; Warnock, 2022). Unicorns – start-ups with a valuation of more than $1 billion – are the most successful scale-ups. As of June 2021, Europe had 92 unicorns, worth €253.3 billion, while the USS had 394 unicorns worth €1.2 trillion (Pitchbook, 2021). High valuation at initial public offerings (IPOs), i.e., when private companies first offer stocks and shares for sale to the public, is another relevant indicator: since 2016, the USA had 71 IPOs worth more than $1 billion, while Europe had only 21 (EIF, 2021b).^1^ The low number of scale-up companies is a concern for EU decision-makers, as these companies contribute significantly to innovation and economic growth (Acs*,* et al., 2011; Moules, 2021).

The EU's underperformance in generating scale-ups is widely attributed to the lack of funding for scale-ups from venture capital (VC) investors (Aernoudt, 2017; Duruflé et al., 2018).^2^ European policymakers have long supported the VC market, improving regulation and injecting substantial financial resources. In the 1997–2015 period, government-owned VC initiatives account for 12.5% of European VC deals, while government-sponsored VC funds account for an additional 29.7% (Alperovych et al., 2018).^3^ In 2019, public resources accounted for 20% of the total €15 billion raised by VCs (source: Invest Europe). The rise of business angels, accelerators and the fintech revolution (e.g., crowdfunding), has made it easier for innovative start-ups to secure early-stage funding and survive long enough for their R&D efforts to reach the market (Aernoudt, 2017). However, scale-ups are more mature companies that typically require further and larger financing rounds. An analysis of Europe's top 1,000 start-ups estimates that reaching unicorn status requires between €100–200 million in funding and a ten-year time horizon (McKinsey, 2018).

Few EU-based VCs are able to meet such large financing needs, and later-stage VC financing is much less well developed in the EU than in the USA. The proportion of later-stage investment in total VC funding is 81% in the USA but only 74% in the EU in the first semester of 2021 (source: Dealroom). This funding gap has – to some extent – been filled by foreign investors, which account for a significant proportion of investments in EU scale-ups (73.1% according to Tech.eu, 2019) with potential negative consequences in terms of relocation of jobs, knowledge, revenue streams, and talent (Braun et al., 2019). An association of European scale-ups, the so-called ‘EU Unicorns Group’, suggested that the European Commission (EC) set up a €100 billion EU sovereign tech fund and a €10 billion EU sovereign green tech fund to address this scale-up finance gap (EU Unicorns, 2021). The argument is that such 'mega-funds' would guarantee access to capital for European (deep) technology and innovation-based firms with high-growth potential and the ambition to scale-up. However, considering the already substantial presence of governments in the EU VC landscape, setting up such mega-funds requires careful scrutiny.

To investigate the need for policy intervention on scale-up funding, in late 2021, the EC organised an online seminar attended by 117 stakeholders. Participants included start-ups and scale-up groups, financial intermediaries, academia, and representatives from European institutions. To our knowledge, this was the first time that private and public stakeholders from the supply and demand sides of high-growth financing came together with academics and policymakers to discuss the available evidence on the scale-up gap in Europe and the policy options for addressing the issue. Analysing and summarising the seminar's outcome, this paper timely contributes to the emerging academic debate on the scale-up gap by providing fresh ideas from policymakers and practitioners.

## 2 Equity gaps, scale-up gap, and rationale for policy intervention

Innovative entrepreneurial ventures typically need a series of successively larger funding rounds to reach their full potential and become large corporations. Market imperfections can occur at any development phase giving rise to ‘equity gaps’ (Cosh et al., 2009; Cressy, 2012). The first equity gap is the shortage of equity capital needed by innovative start-ups to survive through the seed and early-growth phases. The ‘second’ or ‘scale-up’ equity gap is the shortage of finance experienced by companies seeing to scale-up to become established businesses that typically operate internationally.

The scientific literature has discussed the appropriateness of government intervention in start-up financing for at least 20 years (Lerner, 2002). Information asymmetries, innovation externalities, and agency costs arguably constitute a market failure, which, on account of the potential contribution of entrepreneurship to innovation and economic growth, has been the justification for major public policy attention to the first equity gap.

In the case of scale-up financing, a well-articulated and substantiated rationale for policy intervention is lacking. However, even companies that successfully survived the first ‘equity gap’ might suffer from market imperfections, especially in emergent industries in which the novelty of an investment opportunity or the lack of familiarity with a new business model hampers the ability of investors to evaluate business options and possibly results in adverse selection. Moreover, innovation externalities (like R&D spillovers, Lerner, 2002) might lead to an under provision of investments in innovative scale-ups with respect to the social optimum. Coordination failures between early-stage investors and later-stage investors might also contribute to the scale-up gap (Murray, 1994).

Understanding the size of the scale-up gap is fundamental to gauge its potential economic impact and necessary to justify policy intervention. Cressy (2012) provides an overview of approaches to document the existence of funding gaps. However, few papers have rigorously quantified them. Only Wilson et al. (2018) specifically focused on the scale-up gap, finding that UK-based high-technology and knowledge-intensive companies face a scale-up gap which increased over the 2006–2013 period, reaching £1.2 billion (0.6% of GDP) in 2013. Typically, European policymakers estimate financing gaps in Europe using the USA as a benchmark. The European Investment Bank (EIB) adopted this approach in specific digital sectors, showing, for instance, an annual equity gap in Europe of the order of €5–10 billion in artificial intelligence and blockchain (EIB, 2021).

Scholars have discussed policy interventions to tackle the difficulties faced by start-ups in raising second- and third-round financing. Aernoudt (2017) calls for a holistic policy, addressing both the start-up and scale-up gap and proposing government-backed debt-financing instruments. Duruflé et al. (2018) discuss public policies for the financing of scale-ups, including funding policies, taxation policies, and financial regulatory policies. Cumming et al. (2018) suggest that to address the scale-up gap, public policy should not only focus on increasing the supply of finance, but on mitigating frictions among different sources of capital to facilitate the scale-up process (e.g., from crowdfunding to angels, VCs, and IPOs).

In this paper, we enrich the current discussion on the existence, causes, consequences, and appropriate policy response in two ways. First, we focus on the EU context, recognising its distinctive context and on the appropriateness of EU-level policy initiatives. Second, considering the emerging nature of the discussion on the scale-up gap, we contribute with observations and experiences directly collected from stakeholders of the scale-up gap in the EU.

## 3 Data and method

To collect insights on the nature, status, and prospects of addressing the scale-up gap, we relied upon an online seminar in which stakeholders could offer relevant data and express their opinion and insights. The seminar was organised by the European Commission's Joint Research Centre (JRC) together with the Directorate General for Research and Innovation (DG RTD) and the European Innovation Council and SME Executive Agency (EISMEA).^4^ Some 160 experts were invited to the seminar and selected through open interviews and personal contacts on the basis of their research expertise in entrepreneurial finance or hands-on practical knowledge on the scale-up gap problem in Europe. Of these, 22 persons were invited to have an active role in the seminar, comprising academics, representatives from the finance supply side, policymakers, representatives of stakeholder groups (EU Unicorns group, EU Innovation Ecosystems Leaders, Women in VC), and national decision-makers (from France, Slovenia, and Spain).

All participants were invited to read, before the seminar, a seven-page background paper written by the seminar organisers explaining the motivation for the seminar and the underlying research project, providing an overview of current research on the topic and setting the objectives to be achieved by the expert seminar. Specifically, the paper pinpointed a number of key issues to be assessed, such as what is the scale-up gap and in what circumstances it is problematic, the causes and the economic consequences arising from the scale-up gap and the instruments currently available to address the scale-up gap and what other interventions might be necessary to bring about desired changes in scale-up finance.

The expert seminar took place on 5 October 2021. A total of 117 experts participated in the event, of which 25% were academics, 37% public administration officials, 8% representatives of the demand-side actors (e.g., scale-ups and associations), 20% managers of private and public VC funds, and 10% from other organisations, such as start-up accelerators. 38% were female (62% male). The participants represented 25 nationalities – mostly Europeans and a few non-Europeans (USA and Canada).

The seminar was structured thematically, whereby experts presented their views, which were subsequently discussed within the panel and the audience. Half of the session time was reserved for discussion, giving preference to the audience, which was invited to provide comments, concerns, suggestions, and other contributions via the chat channel.

In each session of the workshop, experts discussed an assigned topic led by a moderator and monitored by a non-participating observant. Similar to a focus group methodology, the moderators followed a structured discussion, guided by key questions to be analysed by the participants. The speaker's presentations and the audience contributions in the online chat were collected for further processing. Two academics (co-authors of this paper) acted as rapporteurs of the seminar. They independently drafted a report based on the data collected (minutes, presentations, and chat transcription), verified the participant's statements with the academic literature and other publicly accessible sources, and complemented results with their own insights. A third author merged the two documents into a draft final report. The report was sent to the seminar participants for external validation. Comments received were integrated into a final version (Quas et al., 2021).

The process of data analysis involved the identification and validation by the authors of pertinent themes by monitoring recurring keywords. A text analysis software was used to screen all the conference material (rapporteur notes, slides presented, and record of the seminar online chat) to ensure that all the relevant information was taken into account in the final report. Table 1 provides the list of themes and examples of related keywords.

**Table 1:** Themes identified in the seminar discussion of the scale-up gap.

	Theme	Keywords examples/subthemes.
Causes	Supply	VC funds and fundraising (fund size, immaturity, limited partners' patience and risk appetite, origin of the funds), VC investors (patient capital, risk appetite, low skills).
	Demand	Culture, risk-taking, investor readiness, loss of control, financial knowledge
	Context (ecosystem, framework)	Geographic dispersion, fragmentation of regulation and policy, cultural heterogeneity, lack of EU-wide initiatives, flow of talents and capital, stock markets
Consequences	Threat of relocation	Foreign investors, foreign acquisitions, incubating foreign unicorns
	Vicious cycles	Consequences for early stage, loss of stability, fragmentation
	Economic impact	Lack of growth, jobs, innovation, loss of talent, low productivity
	Strategic consequences	Green Deal, industry standards, data protection
Policy solution	Increase supply	Mega-fund, funds-of-funds, crowding out
	Lessons from academia	Academic evidence, best practices
	Involve private investors	Leverage private, anchor investor, patient investor, incentivise private, blended finance, tax breakers
	Focus the effort	Deep-tech, minorities, emerging economies
	Leverage and improve existing policies	EIC, EIF, InvestEU, ESCALAR, cooperation EU and nations, cut red tape, disincentives for relocation
	Alternatives to VC	Venture debt, corporations, banks
	Increase demand	Public procurement, talent attraction, ‘feeding the pipeline’
	Improve ecosystem	Infrastructure, collaboration, regulation harmonisation, join forces, regulation, flow of capital, flow of talent

## 4 Findings

We summarise the main themes that emerged during the seminar and complement them with the extant VC literature. Where pertinent, we include anonymised quotes from the participants.

### 4.1 Causes of the scale-up gap

While there seems to be general agreement on the existence of a scale-up gap, views diverged on the causes, which we group into the following three factors.

#### 4.1.1 Supply-side factors

**VCfundsand fundraising.** Representatives of the supply side confirmed the general opinion that ‘*European late-stage VC is lacking scale to compete with the USA*’. According to EIF internal data (May 2021), the number of VC funds larger than €500 million was 6–8 times higher in the USA than in the EU. The European market is much ‘*younger and less developed’* than the US market. Younger VCs tend to raise smaller funds, as the average fund size increase with additional fundraising cycles. In Europe, it is also considered more difficult to *fundraise*, especially from pension funds, which are much less at ease in investing in the VC asset class than in the USA. According to Arundale (2019), this is partially due to historical lower VC returns in Europe, which he attributes to the smaller fund size in Europe, less operational and entrepreneurial experience of the limited partners, and their lower propensity to take risks. EIF representatives highlighted that the performance of European VCs improved markedly over the past decade, and as a result, there has been a rapid increase in investment into European VC funds by institutional investors. According to EIF internal data, pension funds increased their investment in VC by 3.5 times between 2015 and 2021. However, their investment in VC remains small – less than 30 times lower than what they invest in private equity. Similar patterns emerge for other categories of institutional investors, such as insurance companies. Governments remain an important source, accounting for 19.7% of funding in the VC industry in 2018–2019 (Atomico, 2020). However, public policies remain largely national, investing resources to seed the local VC industry. This dispersion of policy effort across countries has resulted in a large number of small funds across member states (Duruflé et al., 2018). EU-level support has served to professionalise and develop the VC market over the past decade, but so far has been insufficient to create funds with large financial resources.

**VC investors.** Scale-ups are risky and require long-term investments. VCs organise resources in closed-end funds, which have a limited life, normally 10 years, during which the general partners need to select companies, nurture them, and realise a return for their investors. The investment duration is shorter – typically in the 5–7 years range. These circumstances give the VC an urgency to exit, and a tendency to avoid investments which require long R&D processes (such as biotech, and, more recently, deep-tech). There is no evidence, however, that European VCs are less patient than USA VCs. But there is some evidence of the higher risk appetite of USA-based VCs (Bertoni et al., 2015). Lastly, a point was raised during the seminar regarding the possibly limited screening and mentoring *skills* of EU-based VCs, which might make them less capable of selecting or nurturing promising ventures than their more experienced USA-based peers (Arundale, 2019), and contributes to the limited number of companies ready to scale-up.

#### 4.1.2 Demand-side factors

On the demand side, the paucity of scale-up investments in Europe is linked to a relative low number of high-quality start-ups requiring scale-up funding, which limits investment opportunities for later-stage VCs. Results from the EIF 2021 survey confirm this view, highlighting the increasing competition among investors for potential targets (EIF, 2021a). The small number of potential scale-ups is the result of two factors.

First is the low number of companies that seek external finance. Quas and D'Adda (2018) find that 60% of European high-tech entrepreneurs in their sample do not look for external equity because they think that it is not needed, 10% feel that their demand for funds would be rejected, 13% are concerned with receiving unfavourable financing conditions, and 15% fear of losing control of their business. More educated entrepreneurs have higher chances to look for external finance. Similarly, Eckhardt et al. (2006) find that Swedish start-up entrepreneurs with a higher expectation of market and company growth, and with more industry experience, are more likely to seek external finance. Cosh et al. (2009) find that in the UK, the growth ambitions of entrepreneur firms are linked with their search for VC.

Second is the small number of those companies seeking finance that are selected by investors (typically 1–2%). Scholars, who have investigated the VC selection process (e.g., Gompers et al., 2020), find that common screening criteria by VCs include the prospects for market growth and size, product offerings, the expected rate of return, and the expected risk of a venture project. There are no studies that compare the success rates of companies in Europe that seek VC with those of other major economies. However, several themes emerged during the seminar as potential constraints on the demand for scale-up financing in the EU. First, the *entrepreneurial culture* of European companies differs from that of their US counterparts, in terms of both lower risk attitude and entrepreneurial orientation and reluctance to share control of the business. Second, the *financial knowledge and capabilities* of companies play an important role in successfully raising external finance. It was suggested that innovative start-ups might specifically lack knowledge of the different financing opportunities that are available or may be inhibited from seeking them if they feel they do not fully comprehend their implications. Lastly, *investor readiness* is seen as an important factor. Even high-quality start-ups may be rejected by investors if they are considered not ‘ready’ for funding, in terms of presentation, availability of key financial information on the business, and familiarity with VC contractual details and terminology.

#### 4.1.3 Context factors

*‘**Strong**ecosystems and strong start-ups go hand in hand’.* For many participants, successful scale-up companies not only require funding but also a sophisticated entrepreneurial ecosystem, which encompasses several categories of actors, besides VCs and entrepreneurial ventures, and operates within a coherent and homogenous regulatory framework.

**Geographical dispersion.** The EU entrepreneurial ecosystem is, by nature, *geographically dispersed*. While a handful of VC hotspots characterise the VC industry in the USA (San Francisco, New York, and Boston) and China (Beijing, Shanghai, Shenzhen, and Hong Kong), the European VC industry comprises multiple smaller hubs (Colombo et al., 2019). The geographical distance between investor and investee is detrimental for selection and monitoring, at least at early stages (Cumming & Dai, 2010), but it can be overcome with better infrastructures (Bernstein et al., 2016). Successful entrepreneurial ecosystems also require physical proximity among new and established companies, financial institutions, universities, and incubators (Stam, 2015).

**Fragmentation.** The EU ecosystem is also highly fragmented. Besides geographical distance, *cultural, institutional, and regulative distances* are important barriers to the creation of a pan-European ecosystem for scale-ups. This lowers the chances of start-ups to secure the financial resources (i.e., cross-border investing) and the human capital (i.e., international recruitment) they need to successfully scale-up and limits the options to expand and grow internationally. Moore et al. (2015) find that in the 1996–2005 period, increased normative and cultural-cognitive distance reduced VC investments across European countries, while, interestingly, regulative distance played no role. This concern of fragmentation in Europe also applies to *stock markets*. The fragmented European stock markets are less liquid and provide fewer exit possibilities for VCs than in the USA, as suggested by the smaller number of VC-backed IPOs (2.6% in Europe vs 16.4% in the USA in 2015, Duruflé et al., 2018).

### 4.2 Consequences of the scale-up gap

**Threat of relocation.** An important consequence of the reduced ability of EU VCs to tackle scale-up financing is that *foreign investors* have become dominant in large later-stage VC rounds in European start-ups and scale-ups. According to the Pitchbook data, the total value of European deals with the US investor participation grew 19.4% year-on-year in the period 2011–2020, reaching €23.0 billion in 2020, and out of the 24 VC investors that participated in investments in EU-headquartered unicorns in this period only eight are located in the EU. Such evidence shows that there are good scale-up investment opportunities in Europe that European VC do not pursue. It is suggested this might, at least in part, reflect the fact that from the perspective of US VCs, the lower valuation of Europe-based start-ups makes them an attractive investment alternative to their more highly valued US-based counterparts. From the EU perspective, foreign investments represent a threat because they are very likely to result in *foreign acquisitions*. There is little research on the consequences of the acquisition of entrepreneurial companies (Duncan & Mtar, 2006; Wennberg & Mason, 2017). It is clear that accessing the financial, managerial, and strategic resources of their new owner will enable acquired firms to undergo significant growth. But it can also be argued that acquisition could have less favourable outcomes. For example, some functions may be eliminated because they duplicate those undertaken by the acquiring company or may be relocated to the new owner's corporate location. Regardless of the outcome, it deprives the EU of potential growing EU-based companies. As one of the participants pointed out, *‘Europe cannot become the incubator for US and Asia unicorns, paid by EU taxpayers’*.

**Vicious cycles.** The lack of opportunities for scaling up implies that early investors have lower chances of realising high returns. This reduces the scale of reinvestment by internal and external shareholders and slows the development of the VC industry in the EU as a whole.

**Economic impact.** The lack of late-stage investment has significant adverse consequences on the ability of Europe's successful entrepreneurial businesses to achieve their full potential. First, if left unfinanced, European start-up companies are likely to lack the scale to compete effectively with their international peers. The long-term consequence is the loss of jobs, innovation, productivity, and economic growth that these companies have the potential to generate. Second, in cases of relocation abroad, foreign economies will benefit from those additional jobs and growth at the expenses of the EU. Besides loss of jobs, the scale-up gap also implies a loss of talent. The human capital developed in start-ups, especially those in emerging sectors, is likely to be lost or attracted elsewhere due to the EU scale-up gap if the EU is unable to develop a functioning European ecosystem capable of ‘recycling’ these skills in other companies or activities.

**Strategic consequences.** The lack of scale-up finding might have strategic consequences for the EU. Leading companies in emerging high-tech sectors are likely to set the *industry standards.* Being unable to nurture such leaders in Europe (or forcing them to relocate elsewhere) implies that industry standards will be decided outside Europe, compromising the EU strategic autonomy. Examples include the digital sectors, where scale-up gap might lead to *data protection issues*, and the deep green sectors, where it might compromise the implementation of *European Green Deal*.

### 4.3 Policy solutions for addressing the scale-up gap

There is no simple solution to resolving the scale-up financing gap. The discussion identified the need for an integrated set of policies that addressed the supply side, demand, and the ecosystem.

#### 4.3.1 Increase supply

Many of the representatives of both the demand side (i.e., entrepreneurs) and the supply side (i.e., VC investors and associations) argued that much more public money is needed to address the scale-up gap. Specifically, they called for the creation of ‘mega-funds’ or special ‘funds-of-funds’ to address the issue in particular market segments. Academics and policymakers often mentioned that increasing supply is indeed the ‘“*easiest” policy lever to call for (if not always to deliver on)’* but that evidence for their potential success is limited.

Academic representatives encouraged policymakers to take into account the large body of *academic literature* on the conditions under which funding policies have been successful in supporting start-ups and their design features. One important distinction is made between direct injection of resources in companies and indirect support through private VCs (Alperovych et al., 2018). Governmental VC (GVC) programmes are an example of direct support which has been criticised in light of the negligible (if not negative) impact on the performance of the supported companies (Brander et al., 2015; Cumming et al., 2017). Such underperformance is usually attributed to misallocations by GVC, especially those due to political interferences (Lerner, 2002). However, a potential advantage of the GVC initiatives is that they could attract VC investments in specific companies, such as those facing the scale-up gap, or specific industries and regions (Kovner & Lerner, 2015; Bertoni et al., 2019). Evidence shows that GVCs tend to perform better when they syndicate with private VCs, specialise in specific industries, and adopt a national rather than a regional approach (Alperovych et al., 2020; Cumming et al., 2017). Lerner (2002) discusses the successful USA Small Business Innovation Research (SBIR) programme, established in 1982, which directly funds R&D projects in small high-tech firms. Recent academic evidence points to an evolution of the government intervention in the VC ecosystem, where governments are gradually shifting from the direct GVC approach to the indirect funds-of-funds or co-investment approach, in which ‘Government act as Limited Partners’ (GLP). GLPs follow a ‘market-driven’ approach, where the government provides resources to VC funds managed by private general partners. Previous empirical studies find positive impacts of GLPs on the performance of target companies (Buzzacchi et al., 2013; Brander et al., 2015) suggesting that the indirect GLP approach seems to be preferred to the GVC. Irrespective of the design choice, supply-side initiatives should be considered carefully because they run the risk of ‘crowding out’ private investments (Brander et al., 2015).

**Involve private investors.** To avoid crowding out and considering the EU budget constraints, public funds should not be responsible for the large capital requirements of scale-ups alone. The recommendation is to involve the private sectors as much as possible. Specifically, the government should play the critical *anchor investor* role for VC investors, helping them to attract further resources from private LPs. By doing so, the government could ease the fundraising activity of VCs, leveraging the co-investments of private LPs. But this is possible only if the government is selective in the provision of funds to VC general partners. Moreover, it is important that governments act as patient investors and loosen the pressure on achieving returns in the short term, thus increasing holding periods. This is, for instance, the approach of the ‘Patient Capital Fund’ created by the British Business Bank.^5^ It was also suggested that there is still a stigma associated with European VC among institutional investors, which needs to be addressed by improving the information that is available on the improving performance of European VC.

**Incentivise private investments.** GLP initiatives can incentivise pension funds and other major European pools of capital to invest in selected VC funds by adopting asymmetrical limited partnership agreements which improve the risk-return profile for private limited partners. Successful examples of asymmetric limited partnership include the British Business Bank (UK) and the Australian Innovation Investment Funds, where GLP returns are capped and subordinated to those of private LPs. In the USA, the SBIC programme provides guaranteed debt rather than equity to selected VC funds, covering up to two-thirds of the total fund size. The SBA-guaranteed capital is low cost and does not share in the profits. It leverages the investment by private LPs, and, at the same time, LPs benefit from the SBA's careful monitoring of funds' performance and regulatory compliance.

**Focus the effort.** Participants suggested that the EC should focus on categories of companies for which the scale-up gap is particularly acute, namely emerging technologies, and in particular deep-tech and green-tech, minority-lead start-ups, and emerging economies. These types of firms risk of being neglected because of the ‘herding behaviour’ of VC investors, who prefer to invest in familiar industries, such as ICT, geographical areas, such as traditional hubs, and within selected professional and personal networks, the ‘*old boys network*’ (Aernoudt & De San José, 2020; Cumming & Dai, 2010; Gompers et al., 2016). The lack of investment in emerging technologies, peripheral regions, and minority entrepreneurship is particularly troublesome for the EU as they put at risk policy objectives, such as the Green Deal, the Capital Market Union, and social inclusion objectives.

**Alternatives to VC.** Venture debt is increasingly becoming an important complement to conventional VC equity financing. It has been a key factor in the substantial increase in rounds size in the USA (de Rassenfosse & Fischer, 2016). Although less common than in the USA (Duruflé et al., 2018), venture debt activity in Europe has increased by 6–8 times in the last ten years, suggesting a market estimate of $1.5 billion in 2020 (Atomico, 2020). Corporations engaging in strategic investment could also represent a valuable alternative to conventional VC. Corporate VC investors are more patient than traditional VC investors (Guo et al., 2015) and are arguably better equipped for making investments in emerging industries in which they might have a strategic interest. It was noted in the seminar, ‘*Corporates and non-traditional investors are starting to take up the opportunity to some extent*’. Finally, many participants reflected on the role that banks could have in the scale-up process. In summary, the recommendation is to not only focus on VC but also envisage support alternative sources for capital for scale-ups, improving regulation and, if necessary, introducing incentives (such as tax breaks).

**Leverage and improve existing policies.** When representatives of the EC presented and discussed potentially relevant current EU policies to address the scale-up gap, the general recommendation was *‘to build on what we have and leverage existing tools rather than starting to do something new directly’*. Currently, the EU has three lines of actions that are potentially relevant to the scale-up gap. First, the European Innovation Council (EIC) provides patient capital in the form of equity or quasi-equity up to €15 million to very early-stage innovative companies and is intended to invest, especially in emerging technologies – though the EIC work programme for 2022^6^ introduces some new elements of relevance for scale-up financing. Second, under InvestEU, the EU has several programmes targeted to the growth stage, supporting financial intermediaries, such as debt providers (through guarantees) and VC funds (acting like a limited partner). Third, European Scale-up Action for Risk Capital (ESCALAR) is a ‘non-pari-passu’ scheme aiming at attracting private investors in scale-up financing. ESCALAR's investments have different terms to those made by private investors. An investment from ESCALAR can therefore enhance the risk-adjusted returns to LPs, thereby attracting investors to the asset class. Currently, in the pilot phase, this is the only initiative specifically targeted to scale-up investments. Suggestions for improving current policy included reducing the bureaucratisation of the application process for EU support, and a better collaboration between EU and member states to remove national barriers to the acquisition of EU resources. Further, it was suggested that EU policies should monitor the relocation of their supported companies to avoid the waste of EU resources in companies that contribute to foreign development after moving abroad.

#### 4.3.2 Increase demand

Besides increasing the supply of risk capital, EU governments should boost the high-quality demand for scale-up financing, by improving the entrepreneurial culture across the EU, improving financial education, launching mentoring initiatives and investment-ready programmes, and ensuring that potential scale-ups have the possibility to build human and social capital through international recruitment and cooperation. It was suggested that the EU's entrepreneurial successes should be publicised, with successful EU entrepreneurs promoted as role models, to encourage further entrepreneurial efforts.

Further, governments should use their *procurement* to create demand in the market, especially for companies that are working on climate-related deep-tech, similar to NASA and DARPA in the USA. Procurement formats should also seek to lower the barriers to entry for businesses with innovative solutions to societal challenges. The size of government contracts and speed of decision-making are critical features in the design of effective procurement strategies.

Another lever to boost the demand for scale-up is to *feed the pipeline*. There is a need for sufficient quantity and variety of funding sources in the early stages of the funding escalator to ensure that the scale-up funds have a flow of high-quality investment opportunities. This is particularly important in some sectors, notably deep-tech, where businesses take longer to reach the point where they are investable and become profitable.

#### 4.3.3 Improve the ecosystem

Overcoming the fragmentation of the EU was highlighted in the seminar as a fundamental policy step. Interactions between ecosystems are critical to enable entrepreneurs to access resources that are not available in their own ecosystem. This is particularly important for smaller ecosystems. Policymakers, therefore, need to focus on improving the connectivity of ecosystems in different member states. The *harmonisation and simplification* of regulation, taxes, option schemes, bureaucracy, and EU funding programmes are necessary to improve the chances of EU-based start-ups to raise scale-up financing (through cross-border investment) and human capital (through international recruitment) as well as to grow internationally.

**Flow of capital.** Currently, most VC investments are focused in Germany, France, Benelux and the Nordic region, with disproportionally high amounts flowing to their capital cities. The geographical concentration of VC investing, especially later-stage deals, results in a highly uneven geographical pattern of unicorns both between countries and within countries, with a high concentration in a small number of hubs (Paris, Berlin, Stockholm, and Amsterdam). *EU-level* funding *initiatives* which cooperate with national governments are needed to build an efficient, coherent, and well-connected ecosystem for scale-up funding that is easy to access from anywhere in EU.

**Flow of talent.** Access to key workforce skills is deemed as just as important a constraint on firm growth as finance. This is a particular issue for businesses in smaller ecosystems, which may have to relocate from their home location or direct their expansion to other locations, and has been shown to often have negative impacts on their local and regional economies (Brown & Mawson, 2016). Hence, there is a need for instruments that address the *supply of skilled workers*, particularly tech workers. This includes interventions at school and university/tertiary levels and life-long learning as well as incentives for firms to train employees in-house (which helps to mitigate their risks if such workers then move to other businesses). Businesses also need to *‘facilitate easier and faster entry of global talent into Europe’* In China, the state worked on the attraction of talent from abroad, namely from the Silicon Valley, by lowering living cost for returnees and foreign talent (e.g., Shenzhen talent housing) (Tung, 2008). Further, it is important that the EU-nurtured talent is not lost. *Entrepreneurial recycling* means making sure that skilled workers generated by successful businesses continue to contribute to the ecosystem after business acquisition, relocation, or even closure, being recruited by other firms, setting up new businesses, becoming angel investors or establishing entrepreneurial support mechanisms (e.g., accelerators, incubators, and mentoring) (Spigel & Vinodrai, 2021).

## 5 Discussion

This paper presents the findings of an expert consultation process with 117 experts who participated in an online seminar to discuss the scale-up finance gap in the EU. The group represented both the demand and supply sides and was complemented by experienced academics in entrepreneurship and policymakers at national level and from European institutions.

Evidence shows that the EU is lagging behind the USA and China in its ability to transform its innovative start-ups into high-growth companies in general and unicorns, in particular. According to the expert group, the failure to address the financing needs of scale-ups may result in outcomes that are detrimental for Europe. Some EU-born unicorns have already transferred their headquarters abroad or floated their companies on the USA stock markets. The risk in the mid-term is that technologies, knowledge, and jobs are relocated elsewhere and in the long-term that the EU loses share in the global tech market, ultimately compromising its technological sovereignty. In the words of one expert, Europe risks to becoming an ‘incubator’ for other world regions, without being able to harvest the results of publicly funded research programmes.

The EU has a number of policies and instruments to facilitate the funding of small and medium-sized enterprises (SMEs), start-ups, and high-growth enterprises, within programmes such as InvestEU and Horizon Europe. The most relevant programmes for scale-ups are the EIC Fund and ESCALAR, which are, however, insufficient, due to limited size and pilot character. Other measures, such as the SME IPO fund, directed to support the IPO of SMEs, are helpful complementary instruments but not suitable on their own to solve the scale-up gap. This has resulted in calls for the creation of sovereign mega-funds in the order of €100 billion. However, a large majority of the experts highlighted the risk that setting up such public mega-funds would, because of their size, distort the VC market potentially crowding out private investors. The consensus was that public funding should always be matched with private capital, which can be attracted by improving the risk-return profile for private investors using non-pari-passu strategies. Further, public schemes should be made available on a temporary basis until the later-stage VC industry matures and is able to operate independently from public support.

Although adequate funding is indispensable for start-ups to grow, providing funds is not sufficient. Decision-makers need to activate policy levers beyond finance which contribute to the development of scale-ups and future unicorns. These include measures to build supportive entrepreneurial ecosystems, ensuring the presence of favourable framework conditions, such as enhancing the competences of workers, promoting relationships with academia, and other grass-roots measures that nurture the ‘pipeline’ of start-ups with flagship innovative projects and technologies. Of supreme importance to the participants was revising current regulations to reduce the barriers to international expansion, recruitment, and investments.

The expert group also identified several ‘policy dilemmas’ whose analysis falls beyond the scope of this study but that deserve further academic attention. The first dilemma is how to strike the balance between supporting EU ‘champions’ or ‘outliers’ on one hand, and on the other, spreading financial support to a broader range of promising high-growth firm. The majority view of the experts was that public involvement should first support those segments that are less attractive for private investors and at the same time are the most strategic for the EU (the deep-tech sector was a prominent example). However, more academic work is needed to identify which industries, regions, or entrepreneurial profiles are more exposed to the scale-up gap and in greater need of government intervention.

Another dilemma is regarding the tension between addressing regional disparities within the EU and acknowledging the importance of geographical concentration for a well-functioning ecosystem for scale-ups. While the geographical concentration of VC investors around the Silicon Valley has been one of the most important ingredients of the USA's entrepreneurial success, each European government, in the attempt to replicate such success in its own jurisdiction, has contributed to the fragmentation of the VC industry that has resulted in the relatively small size of the European VC funds (Nightingale et al., 2009). EU scale-up funds, equally accessible from any EU nation, are needed.

It is of crucial importance, before taking any policy action, to quantify the scale-up gap and to determine its economic impact in the EU. There is a need for a commonly accepted methodology to assess financing gaps and to analyse whether the scale-up gap is an outcome of too few good companies (i.e., investment opportunities) or too few good investors (i.e., too little funds). The issue is that there is no reliable measure to capture appropriately the demand side of the gap. Most of the demand for scale-ups is ‘latent’ and includes entrepreneurs who choose to make an early exit (via acquisition) in absence of scale-up finance. The individual characteristics of entrepreneurs, such as gender, culture, personality traits, educational and professional background, and industry sector also influence their probability of looking for scale-up finance as well as the amounts required. The recommendation for future research is therefore to base the assessment of the scale-up gap on micro-level data that takes account of the heterogeneity in both the demand and the supply side across sectors, geographical areas, and entrepreneurial profiles, as well as its evolution over time.

How to quantify the economic consequences of the scale-up gap is another largely unaddressed empirical challenge. While employment is a frequently used metric to measure impact, scale-ups also have other macroeconomic influences, notably on innovation and productivity. The potential impact of scale-ups also spills over to other businesses and activities in the ecosystem, such as start-ups, other scale-ups, and financial institutions. To account for such impact, researchers would need to construct comprehensive databases, covering longitudinal data to study the dynamic development of an ecosystem, and complement micro-level studies (i.e., company level) with analysis at the entrepreneurial ecosystem level. Assuming that the scale-up gap exists and has an economic impact, more theoretical work is needed on the analysis of the origin of the market failures that, arguably, are responsible for the gap (and we hope that this paper goes in this direction).

Lastly, several further questions emerge regarding how to best design policies to address the scale-up gap. Studies on the most effective policies addressing the ‘first equity gap’ could serve as a starting point (e.g., Alperovych et al., 2020). In closing, we reiterate that to effectively address the scale-up gap funding policies need to work alongside instruments that encourage demand and improve the ecosystem. This calls for studies that consider ‘scale-up policy mixes’ rather than single policies.

## Funding

Work by C Mason and A Quas was covered under JRC Contract EMI Pool 043129.

## Footnotes

1 The paucity of unicorns and of high-value IPOs in Europe is also influenced by the higher average valuations in the USA with respect to Europe, especially for second and later rounds of financing (Duruflé et al., 2018). A tendency of overvaluation in the USA might, therefore, also explain part of the apparent scale-up gap.

2 As there is no consensus yet on the concept of “scale-ups” in the literature, we adopt the view of Duruflé et al. (2018), who define scale-ups as entrepreneurial ventures that have found their initial product/service offering and market segment and are entering a growth phase where they seek significant market penetration.

3 The presence of governments in VCs is important all over the world: Brander et al. (2015) document that 17% of investments in the USA are funded with public money either through the government-owned or government-sponsored funds.

4 Three of the authors of this paper were in the organizing team, as members of the JRC at the time of the seminar.

5 https://www.britishpatientcapital.co.uk/.

6 https://eic.ec.europa.eu/news/european-innovation-council-biggest-annual-funding-opportunities-innovators-scale-and-capture-global-2022-02-09_en.
